# Intrinsic protein disorder and conditional folding in AlphaFoldDB

**DOI:** 10.1002/pro.4466

**Published:** 2022-10-26

**Authors:** Damiano Piovesan, Alexander Miguel Monzon, Silvio C. E. Tosatto

**Affiliations:** ^1^ Department of Biomedical Sciences University of Padova Padova Italy; ^2^ Department of Information Engineering University of Padova Padova Italy

**Keywords:** CAID, critical assessment, intrinsically disordered proteins, machine learning, protein structure, structural bioinformatics

## Abstract

Intrinsically disordered regions (IDRs) defying the traditional protein structure–function paradigm have been difficult to analyze. The availability of accurate structure predictions on a large scale in AlphaFoldDB offers a fresh perspective on IDR prediction. Here, we establish three baselines for IDR prediction from AlphaFoldDB models based on the recent CAID dataset. Surprisingly, AlphaFoldDB is highly competitive for predicting both IDRs and conditionally folded binding regions, demonstrating the plasticity of the disorder to structure continuum.

The prediction of protein tertiary structure from sequence has been considered the Holy Grail of structural biology since at least the 1960's, with generations of researchers claiming progress. Since 1994, the biennial Critical Assessment of techniques for protein Structure Prediction (CASP) experiment has tried to measure the state‐of‐the‐art and progress in the field. In CASP14,^1^ AlphaFold has at last demonstrated a breakthrough thanks to its clever use of machine learning and multiple sequence alignments.^2^, ^3^ This is leading to a paradigm shift for structural biology due to the sudden availability of orders of magnitude more protein structures.^4^, ^5^ AlphaFoldDB expands the impact further by allowing interested researchers to browse predictions for proteins in several major model organisms.^6^, ^7^ This wealth of information is being used to map out less studied parts of the proteome.^8^, ^9^ It has highlighted the presence of a considerable fraction of the human proteome with low AlphaFold accuracy scores that may reflect intrinsically disordered regions (IDRs) in proteins.^7^, ^10^ IDRs lacking a fixed tertiary structure are well‐known in structural biology^11^ and have been associated with a variety of biological functions.^12^

We have recently described the first round of the Critical Assessment of Protein Intrinsic Disorder (CAID) experiment,^13^ which aims to establish the state‐of‐the‐art for IDRs in a similar way to CASP. CAID provides two separate challenges, each using experimental IDR information that has been manually curated from the literature and deposited in DisProt.^14^ These challenges represent prediction of IDRs (DisProt) and prediction of those IDR sub‐regions responsible for, mostly transient, binding to other molecules (DisProt‐binding). In both cases, positive annotation comes from DisProt and negatives are non‐annotated residues. To account for incompleteness of IDR annotation in DisProt, CAID defines a variant of the disorder prediction dataset (DisProt‐PDB), where the negatives are restricted to residues observed in the Protein Data Bank (PDB),^15^ so that experimentally “uncertain” residues are excluded.

Here, we tested how AlphaFoldDB performs in comparison to other state‐of‐the‐art methods for both disorder and binding categories. From our results, we will draw some interesting conclusions regarding the relationship between IDRs, binding and predicted structures. The assessment was carried out exactly as detailed in CAID.^13^ The AlphaFoldDB predictions were downloaded on July 20, 2021. Only 489 proteins out of the 645 evaluated in CAID were provided in AlphaFoldDB at that time. The code is implemented as a Python script and freely available from https://github.com/BioComputingUP/AlphaFold-disorder. Since AlphaFoldDB stores predictions for protein tertiary structure, we first need to establish how it can be used to predict IDRs. Since we are interested only in a proof‐of‐principle, three simple AlphaFold scores were defined as follows.

The authors of AlphaFold already noted how regions for low predicted accuracy (pLDDT score) are correlated to IDRs.^6^ AlphaFold‐pLDDT uses 1 ‐ pLDDT as output. The optimal classification threshold (0.312, representing pLDDT <68.8%) was selected by maximizing the F1‐Score performance on the CAID DisProt dataset.

Visual inspection shows how AlphaFold tends to predict regions without stable structure as “ribbons” surrounding the folded core (see Figure 1 for an example). Intuitively, these residues share a high solvent accessible surface, which can be used as a proxy to describe the “ribbon” structure. A second disorder definition calculates the relative solvent accessibility (RSA) over a local window centered on the residue to predict (AlphaFold‐RSA). The DSSP solvent accessibility^16^ is normalized for each residue by the maximum accessibility of a fully extended Gly‐X‐Gly peptide^17^ as provided by the BioPython PDB module.^18^ The optimal local window size (25 residues, that is, +/−12), was chosen with a grid search (range: 1–50 residues) resulting in a plateau between 20 and 30 residues. Mirroring is used in the local window for positions close to the sequence termini. The optimal classification threshold for AlphaFold‐RSA (0.581) was again selected by maximizing the F1‐Score performance on the CAID DisProt dataset and should be considered an optimistic upper bound for its performance.

**FIGURE 1:**
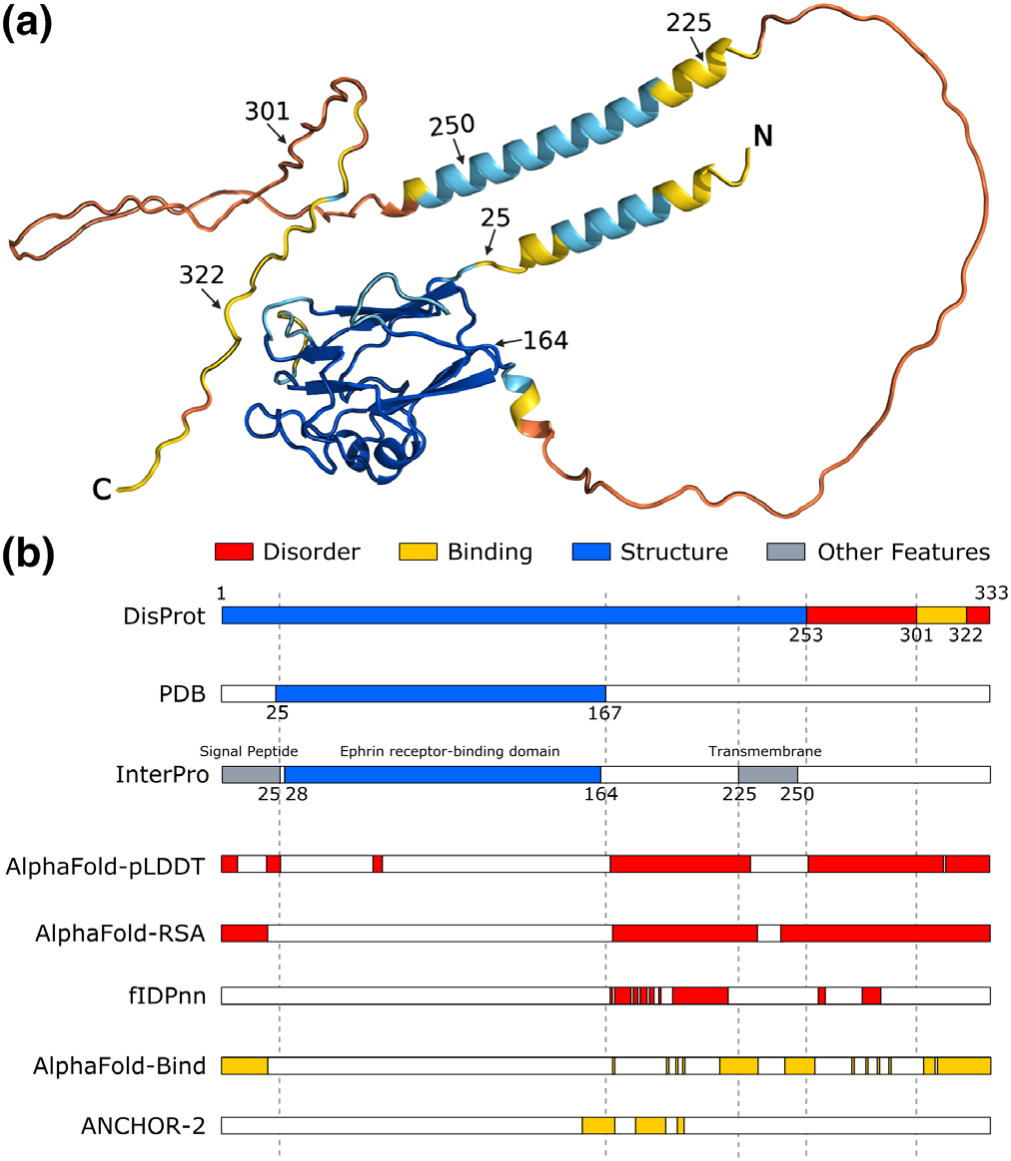
Example of intrinsically disordered regions and conditional folding predictions derived from AlphaFold and best predictors. The human Ephrin‐B2 protein (UniProt accession: P52799) is shown as a representative example to illustrate the overlap between AlphaFold predictions and various sequence features. Panel a, the structure of the protein predicted by AlphaFold and colored by pLDDT score (<50 orange, <70 yellow, <90 light blue, >90 dark blue). Residue labels indicate annotated region boundaries. Panel b, database annotations (DisProt DP01588, PDB, InterPro P52799) and predicted regions (AlphaFold‐pLDDT, AlphaFold‐RSA, AlphaFold‐Bind, fIDPnn,^22^ ANCHOR‐2^19^). PDB annotation is generated by combining observed residues in different PDB experiments. Best predictors were selected based on their performance against DisProt and DisProt‐binding references. Annotated regions are shown colored according to the legend on top of panel b (i.e., disorder in red, binding in gold, structure in blue, other features in gray, while white regions correspond to no annotation. Per‐residue AlphaFold predictions are provided in Figure S1

Visual inspection also shows that some “ribbon” regions have high RSA yet retain some local secondary structure and have a relatively high pLDDT score, suggesting the possibility to act as disordered binding regions. The combination of both features can likewise be used to identify regions with a tendency to be simultaneously accessible (RSA) and structured (pLDDT), which may indicate disordered binding regions or conditional folding (AlphaFold‐Bind). AlphaFold‐Bind combines the previous two scores using the following formula:

$$\text{AlphaFold}\_B\text{in}d\left(T\right)=\left\{\begin{array}{c} \text{AlphaFold}\_\mathrm{RSA},\\ \;\text{AlphaFold}\_\mathrm{RSA}\leq T\\ T+\text{ρLDDT}\left(1-T\right),\\ \;\text{AlphaFold}\_\mathrm{RSA}>T \end{array}\right.$$

with T set to the AlphaFold‐RSA classification threshold (0.581). This ensures values between 0 and 1 by scaling the score for pLDDT accordingly. The optimal classification threshold for AlphaFold‐Bind (0.773) was again selected by maximizing the performance on the CAID DisProt‐Binding dataset. Notice that this is a proof of principle implementation only. Performance can likely be increased using simple measures such as smoothing over a local window.

We have repeated the entire CAID analysis presented previously^13^ by including the three AlphaFoldDB variants described above, with the full results available as Appendix S1. As expected, AlphaFold‐pLDDT performs well on the CAID PDB‐DisProt dataset^6^ (see Figure 2a, Tables S1 and S2). The performance is increased using AlphaFold‐RSA, which has the highest accuracy of all tested IDR prediction methods. As previously noted, this definition suggests that intrinsic disorder can be considered the opposite of globular structure.^13^ When using the DisProt definition, the situation changes somewhat (see Figure 2b, Tables S3 and S4), with AlphaFold‐RSA being among the top five methods. This result can suggest a difference between low AlphaFold prediction confidence (as expressed in AlphaFold‐pLDDT) and intrinsic disorder. Indeed when compared to other methods, both AlphaFold‐RSA and AlphaFold‐pLDDT show a lower precision at low recall (Figure 2c) and a lower TPR at low FPR (Figure 2d), that is when focusing on the left part of the precision‐recall and ROC curves. This indicates that the score does not follow disorder confidence, that is, high score is not predictive of bona fide disorder. When used to identify fully disordered proteins AlphaFold‐pLDDT and AlphaFold‐RSA behave differently, the first under‐predicts and the second over‐predicts (Table S7). AlphaFold‐pLDDT always predicts some residual structure, even if transient.

**FIGURE 2:**
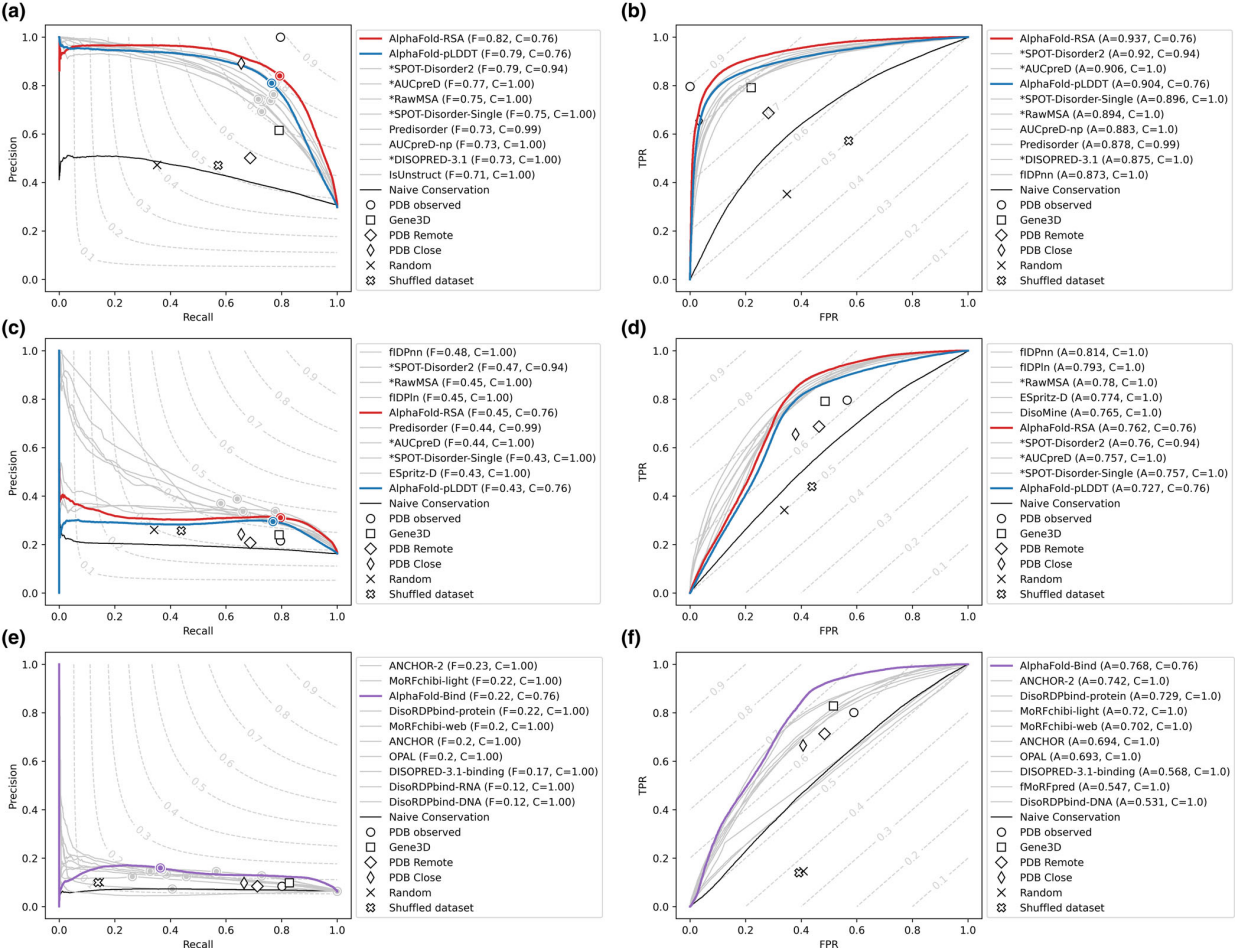
Results for AlphaFold on the three main CAID categories. The results for the DisProt‐PDB (*n* = 646 proteins, panels a,b), DisProt (*n* = 646 proteins, panels c, d), and DisProt‐binding (*n* = 646 proteins, panels e, f) references are shown. Performance of predictors expressed as maximum F1‐Score across all thresholds (*F*_max_) (panels a, c, e) and AUC (panels b, d, f) for AlphaFold (colored), the top 10 best ranking methods (gray) and baselines (white symbols) are shown. The legend on the right of each panel shows the name of the method alongside its *F*_max_ or AUC score (f and a, respectively) and coverage (c). Notice how the latter is usually 1.0 for most predictors, but only 0.76 for AlphaFold as predictions for some targets are not available

Given the good AlphaFold performance in predicting disorder, we wondered whether it could also be used to predict binding regions. Conceptually, AlphaFold is not designed to predict binding regions, being limited to single protein chains and intra/protein contacts.^3^ When the interaction partner is known and the input properly adapted, AlphaFold has been shown to predict the structure of protein complexes with good accuracy.^9^ This is possible because inter/chain contacts between interaction surfaces are well encoded in multiple sequence alignments (and in the model) similarly to intra/chain contacts.

Being able to use AlphaFoldDB to predict disordered binding regions a priori, that is, without knowing the input partner, is therefore not expected. Disorder binding is inherently different from rigid surface‐surface interactions, being transient, often multivalent, and versatile, adapting to a number of diverse partners.^12^ Indeed, both AlphaFold‐pLDDT and AlphaFold‐RSA do not perform well on this task (see Figure 2c, Tables S5 and S6). Strikingly, the combination of both scores (AlphaFold‐Bind) performs very well and reaches state‐of‐the‐art performance on par with ANCHOR2^19^ on the DisProt‐Binding dataset.

The ability of AlphaFoldDB to predict IDR binding regions is not entirely surprising. It has been known for a while that binding often occurs in parts of the sequence which have a tendency for disorder close to the decision threshold. This is at the basis of methods such as ANCHOR.^20^ Intuitively, the definition we use for AlphaFoldDB falls in the same mold. High solvent accessibility implies the lack of overall structure, while a higher pLDDT score implies some residual local structure. From a biophysical perspective, disorder and secondary structure are both encoded in the protein sequence.^21^ As AlphaFold leverages large sequence alignments to generate its models,^3^ it is implicitly encoding sequence conservation in its predictions and this is reflected in the pLDDT score. Hence, it is logical to be able to identify regions undergoing conditional folding.

On the other hand, AlphaFoldDB currently does not provide a thorough description of the structural ensemble for IDRs.^10^ The relative movements of residues cannot be captured with a single static structure. AlphaFold‐pLDDT fails in identifying fully disordered proteins as some residual structure is always predicted, even if transient (Table S7). Being able to recognize conditional folding events by combining pLDDT and solvent accessibility can however help distinguish in a more coarse grained manner the relative rigidity of the polypeptide chain, separating spacer regions from those involved in transient binding.

Finally, the execution time of AlphaFold is two orders of magnitude slower than methods with similar performance,^13^ indicating AlphaFold currently is not the best choice for fast searches at the genomic scale.

We have shown how AlphaFoldDB can be used both to predict IDRs as well as the binding regions inside them. It should be cautioned however that this is not a true blind test and may overestimate its performance. We look forward to being able to assess AlphaFoldDB and similar methods fully as part of the next round of CAID.

## AUTHOR CONTRIBUTIONS

**Damiano Piovesan:** Conceptualization (equal); methodology (equal); software (equal); writing – original draft (equal); writing – review and editing (supporting). **Alexander Miguel Monzon:** Investigation (equal); resources (equal); software (equal); writing – original draft (supporting); writing – review and editing (supporting). **Silvio C E Tosatto:** Conceptualization (lead); funding acquisition (lead); methodology (equal); supervision (lead); writing – original draft (equal); writing – review and editing (lead).

## CONFLICT OF INTEREST

The authors declare no conflict of interest.

## Supporting information


**Appendix S1** Supporting information.

## Data Availability

The data used including AlphaFoldDB predictions is available from the URL https://idpcentral.org/caid/data/1_alphafold/. All data used in the analysis are also available in the Code Ocean capsule available at URL https://doi.org/10.24433/CO.6770815.v1. The CAID assessment can be fully reproduced downloading the code and following the instructions in the CAID GitHub repository at URL https://github.com/BioComputingUP/CAID. The code is also available and reproducible in the Code Ocean capsule available at URL https://doi.org/10.24433/CO.6770815.v1. The code to generate AlphaFoldDB disorder predictions is implemented as a Python script and available at URL https://github.com/BioComputingUP/AlphaFold-disorder.
