# Supplementary material for: Intrinsic protein disorder and conditional folding in AlphaFoldDB
Source: Protein Sci. 2022 Oct 26;31(11):e4466. doi: 10.1002/pro.4466 (PMC9601767; doi:10.1002/pro.4466)
Supplement: Supplementary file 1 — Appendix S1 Supporting information. [file PRO-31-e4466-s001.pdf]

Intrinsic Protein Disorder and Conditional Folding in AlphaFoldDB

*Damiano Piovesan, Alexander Miguel Monzon, Silvio C.E. Tosatto**
Dept. of Biomedical Sciences, University of Padova, Italy.

Supplementary Information

Table of Contents

DisProt-PDB dataset **2**
Supplementary Table 1. Per-residue classification performance in the DisProt-PDB dataset. 3
Supplementary Table 2. Per-protein classification performance in the DisProt-PDB dataset. 4

DisProt-dataset **6**
Supplementary Table 3. Per-residue classification performance in the DisProt dataset. 7
Supplementary Table 4. Per-protein classification performance in the DisProt dataset. 8

DisProt-Binding dataset **10**
Supplementary Table 5. Per-residue classification performance in the DisProt-Binding dataset. 10
Supplementary Table 6. Per-protein classification performance in the DisProt-Binding dataset. 11

Fully Disordered Proteins **12**
Supplementary Table 7. Confusion matrix and metrics for the prediction of fully disordered proteins in the DisProt dataset. 13
Supplementary Table 8. AlphaFold-RSA performance of predicted fully disordered proteins. 15

Figures 16
Supplementary Figure 1 - Intrinsic disorder and conditional folding per-residue scores derived from AlphaFold prediction. 16

DisProt-PDB dataset

	BAC	F1-s	FPR	MCC	PPV	TPR	TNR	COV
**PDB observed**	**0.898**	**0.886**	**0.000**	**0.854**	**1.000**	**0.796**	**1.000**	**646**
AlphaFold-RSA	*0.862*	*0.816*	*0.058*	*0.742*	*0.851*	*0.783*	*0.942*	*489*
*SPOT-Disorder2	0.848	0.792	0.078	0.705	0.811	0.773	0.922	610
AlphaFold-pLDDT	*0.842*	*0.786*	*0.070*	*0.701*	*0.821*	*0.753*	*0.930*	*489*
*AUCpreD	0.831	0.767	0.108	0.660	0.763	0.771	0.892	644
**PDB Close**	**0.811**	**0.755**	**0.033**	**0.689**	**0.891**	**0.655**	**0.967**	**604**
*SPOT-Disorder-Single	0.822	0.753	0.118	0.640	0.743	0.763	0.882	646
*RawMSA	0.819	0.749	0.119	0.635	0.740	0.758	0.881	646
AUCpreD-np	0.804	0.731	0.115	0.612	0.738	0.724	0.885	646
Predisorder	0.802	0.730	0.108	0.612	0.748	0.712	0.892	642
*DISOPRED-3.1	0.802	0.729	0.108	0.612	0.746	0.712	0.892	646
fIDPnn	0.792	0.710	0.150	0.574	0.686	0.735	0.850	645
IsUnstruct	0.790	0.710	0.133	0.579	0.706	0.714	0.867	646
VSL2B	0.789	0.707	0.139	0.574	0.696	0.718	0.861	644
IUPred2A-long	0.785	0.706	0.110	0.582	0.733	0.680	0.890	646
MobiDB-lite	0.783	0.704	0.106	0.583	0.739	0.673	0.894	645
IUPred2A-short	0.787	0.704	0.142	0.569	0.692	0.717	0.858	646
ESpritz-X	0.783	0.699	0.138	0.563	0.694	0.704	0.862	645
DisoMine	0.782	0.695	0.167	0.550	0.661	0.732	0.833	646
ESpritz-D	0.784	0.694	0.199	0.542	0.633	0.767	0.801	645
**Gene3D**	**0.785**	**0.692**	**0.220**	**0.539**	**0.615**	**0.791**	**0.780**	**646**
fIDPln	0.777	0.687	0.183	0.536	0.642	0.738	0.817	645
ESpritz-N	0.767	0.681	0.121	0.546	0.707	0.656	0.879	645
JRONN	0.771	0.681	0.163	0.532	0.659	0.704	0.837	645
DynaMine	0.753	0.656	0.192	0.490	0.618	0.698	0.808	645
PyHCA	0.749	0.652	0.180	0.488	0.627	0.678	0.820	646
FoldUnfold	0.736	0.636	0.193	0.462	0.608	0.666	0.807	621
DisEMBL-465	0.709	0.598	0.216	0.406	0.566	0.633	0.784	644
*S2D-2	0.706	0.596	0.292	0.385	0.517	0.704	0.708	644
**PDB Remote**	**0.703**	**0.579**	**0.282**	**0.376**	**0.501**	**0.687**	**0.718**	**523**
DisEMBL-HL	0.640	0.536	0.481	0.262	0.413	0.762	0.519	644
GlobPlot	0.656	0.535	0.294	0.295	0.479	0.605	0.706	645
*DisPredict-2	0.620	0.514	0.469	0.222	0.403	0.708	0.531	646
**Conservation**	**0.614**	**0.508**	**0.473**	**0.212**	**0.398**	**0.701**	**0.527**	**646**
DFLpred	0.498	0.471	1.000	-0.043	0.308	0.996	0.000	646

Supplementary Table 1. Per-residue classification performance in the DisProt-PDB dataset. Performance of predictors and baselines for the *DisProt-PDB* dataset. Metrics are averaged over the whole dataset, sorted by F1-Score and predictor thresholds are optimized on F1-Score. Baselines are shown in bold and AlphaFold in italics and underlined. The evaluation metrics are as follows: balanced accuracy (BAC), F1-Score (F1-s), false positive rate (FPR); Matthews correlation coefficient (MCC), positive predictive value (PPV, i.e. precision), true positive rate (TPR, i.e. recall), true negative rate (TNR, i.e. specificity) and coverage (COV), i.e. number of predicted target proteins (out of 646).

	BAC	F1-s	FPR	MCC	PPV	TPR	TNR	COV
**PDB observed**	**0.817**	**0.751**	**0.260**	**0.426**	**0.847**	**0.844**	**0.740**	**646**
AlphaFold-pLDDT	*0.766*	*0.655*	*0.104*	*0.398*	*0.734*	*0.678*	*0.896*	*489*
AlphaFold-RSA	*0.769*	*0.659*	*0.142*	*0.380*	*0.745*	*0.690*	*0.858*	*489*
*SPOT-Disorder2	0.776	0.666	0.140	0.358	0.728	0.707	0.860	610
**PDB Close**	**0.716**	**0.624**	**0.291**	**0.341**	**0.780**	**0.712**	**0.709**	**604**
*AUCpreD	0.770	0.656	0.174	0.336	0.691	0.726	0.826	644
*DISOPRED-3.1	0.719	0.597	0.132	0.310	0.702	0.621	0.868	646
Predisorder	0.727	0.607	0.170	0.299	0.669	0.658	0.830	642
*RawMSA	0.746	0.623	0.198	0.299	0.683	0.690	0.802	646
AUCpreD-np	0.738	0.617	0.183	0.296	0.663	0.678	0.817	646
*SPOT-Disorder-Single	0.743	0.620	0.207	0.287	0.664	0.690	0.793	646
MobiDB-lite	0.707	0.582	0.177	0.268	0.656	0.621	0.823	645
ESpritz-X	0.722	0.594	0.220	0.268	0.634	0.677	0.780	645
IUPred2A-short	0.724	0.598	0.211	0.268	0.641	0.671	0.789	646
IsUnstruct	0.717	0.597	0.213	0.264	0.643	0.661	0.787	646
VSL2B	0.709	0.589	0.214	0.262	0.641	0.651	0.786	644
ESpritz-N	0.690	0.555	0.165	0.260	0.646	0.594	0.835	645
JRONN	0.705	0.577	0.213	0.254	0.632	0.646	0.787	645
**Gene3D**	**0.740**	**0.625**	**0.380**	**0.243**	**0.657**	**0.778**	**0.620**	**646**
fIDPnn	0.729	0.607	0.311	0.243	0.630	0.731	0.689	645
IUPred2A-long	0.685	0.548	0.184	0.241	0.664	0.585	0.816	646
DynaMine	0.698	0.565	0.233	0.236	0.603	0.647	0.767	645
fIDPln	0.718	0.593	0.361	0.219	0.623	0.740	0.639	645
PyHCA	0.682	0.543	0.258	0.215	0.606	0.632	0.742	646
DisoMine	0.696	0.563	0.333	0.205	0.618	0.686	0.667	646
DisEMBL-465	0.659	0.530	0.265	0.197	0.576	0.613	0.735	644
*S2D-2	0.666	0.526	0.317	0.191	0.561	0.654	0.683	644
**PDB Remote**	**0.664**	**0.521**	**0.349**	**0.185**	**0.595**	**0.651**	**0.651**	**523**
FoldUnfold	0.665	0.553	0.330	0.176	0.618	0.637	0.670	621
GlobPlot	0.622	0.486	0.305	0.152	0.549	0.576	0.695	645
ESpritz-D	0.684	0.552	0.415	0.151	0.569	0.712	0.585	645
DisEMBL-HL	0.663	0.533	0.533	0.131	0.513	0.771	0.467	644
**Conservation**	**0.607**	**0.468**	**0.432**	**0.129**	**0.538**	**0.636**	**0.568**	**646**
*DisPredict-2	0.621	0.496	0.600	0.053	0.495	0.723	0.400	646
DFLpred	0.645	0.549	0.999	-0.001	0.470	0.997	0.001	646

Supplementary Table 2. Per-protein classification performance in the DisProt-PDB dataset. Performance of predictors and baselines for *DisProt-PDB* dataset. Metrics are averaged over the whole dataset, sorted by F1-Score and predictor thresholds are optimized on F1-Score. Baselines are shown in bold and AlphaFold in italics and underlined. The evaluation metrics are as follows: balanced accuracy

(BAC), F1-Score (F1-s), false positive rate (FPR); Matthews correlation coefficient (MCC), positive predictive value (PPV, i.e. precision), true positive rate (TPR, i.e. recall), true negative rate (TNR, i.e. specificity) and coverage (COV), i.e. number of predicted target proteins (out of 646).

DisProt-dataset

	BAC	F1-s	FPR	MCC	PPV	TPR	TNR	COV
fIDPnn	0.719	0.483	0.186	0.369	0.394	0.624	0.814	645
*SPOT-Disorder2	0.722	0.470	0.325	0.345	0.338	0.769	0.675	610
fIDPln	0.691	0.452	0.179	0.329	0.378	0.561	0.821	645
AlphaFold-RSA	*0.723*	*0.447*	*0.332*	*0.336*	*0.313*	*0.779*	*0.668*	*489*
*RawMSA	0.703	0.446	0.248	0.323	0.338	0.654	0.752	646
*AUCpreD	0.709	0.436	0.350	0.315	0.304	0.768	0.650	644
Predisorder	0.686	0.435	0.257	0.298	0.332	0.629	0.743	642
*SPOT-Disorder-Single	0.701	0.433	0.298	0.308	0.313	0.700	0.702	646
ESpritz-D	0.682	0.431	0.215	0.301	0.343	0.578	0.785	645
AlphaFold-pLDDT	*0.701*	*0.426*	*0.341*	*0.303*	*0.298*	*0.744*	*0.659*	*489*
AUCpreD-np	0.688	0.426	0.270	0.295	0.317	0.647	0.730	646
DisoMine	0.691	0.425	0.288	0.295	0.311	0.669	0.712	646
IUPred2A-short	0.683	0.420	0.269	0.287	0.314	0.634	0.731	646
MobiDB-lite	0.678	0.420	0.236	0.288	0.326	0.592	0.764	645
IsUnstruct	0.682	0.418	0.276	0.284	0.310	0.640	0.724	646
ESpritz-X	0.686	0.418	0.299	0.286	0.303	0.672	0.701	645
IUPred2A-long	0.681	0.417	0.278	0.283	0.309	0.640	0.722	646
VSL2B	0.678	0.409	0.303	0.273	0.296	0.659	0.697	644
JRONN	0.661	0.401	0.242	0.260	0.311	0.563	0.758	645
ESpritz-N	0.663	0.400	0.262	0.258	0.303	0.587	0.738	645
*DISOPRED-3.1	0.671	0.394	0.370	0.255	0.272	0.712	0.630	646
PyHCA	0.655	0.386	0.309	0.239	0.280	0.619	0.691	646
DynaMine	0.652	0.385	0.288	0.237	0.285	0.592	0.712	645
**Gene3D**	**0.653**	**0.368**	**0.486**	**0.226**	**0.240**	**0.791**	**0.514**	**646**
DisEMBL-465	0.633	0.367	0.255	0.214	0.283	0.520	0.745	644
FoldUnfold	0.642	0.365	0.382	0.211	0.251	0.666	0.618	621
**PDB Close**	**0.637**	**0.353**	**0.380**	**0.202**	**0.242**	**0.655**	**0.620**	**604**
*S2D-2	0.623	0.347	0.423	0.181	0.234	0.668	0.577	644
**PDB observed**	**0.616**	**0.339**	**0.565**	**0.174**	**0.215**	**0.796**	**0.435**	**646**
DisEMBL-HL	0.605	0.333	0.312	0.162	0.244	0.522	0.688	644
*DisPredict-2	0.601	0.329	0.368	0.152	0.231	0.569	0.632	646
GlobPlot	0.592	0.321	0.415	0.137	0.219	0.600	0.585	645
**PDB Remote**	**0.611**	**0.318**	**0.464**	**0.159**	**0.207**	**0.687**	**0.536**	**523**
**Conservation**	**0.547**	**0.294**	**0.682**	**0.075**	**0.181**	**0.776**	**0.318**	**646**
DFLpred	0.498	0.279	0.998	-0.032	0.162	0.994	0.002	646

Supplementary Table 3. Per-residue classification performance in the DisProt dataset.
Performance of predictors and baselines for *DisProt* dataset. Metrics are averaged over the whole dataset, sorted by F1-Score and predictor thresholds are optimized on F1-Score. Baselines are shown in bold and AlphaFold in italics and underlined. The evaluation metrics are as follows: balanced accuracy (BAC), F1-Score (F1-s), false positive rate (FPR); Matthews correlation coefficient (MCC), positive predictive value (PPV, i.e. precision), true positive rate (TPR, i.e. recall), true negative rate (TNR, i.e. specificity) and coverage (COV), i.e. number of predicted target proteins (out of 646).

	BAC	F1-s	FPR	MCC	PPV	TPR	TNR	COV
*SPOT-Disorder2	0.704	0.481	0.306	0.306	0.455	0.702	0.694	610
AlphaFold-RSA	*0.689*	*0.455*	*0.317*	*0.303*	*0.444*	*0.685*	*0.683*	*489*
AlphaFold-pLDDT	*0.691*	*0.435*	*0.283*	*0.303*	*0.423*	*0.667*	*0.717*	*489*
*AUCpreD	0.698	0.461	0.340	0.281	0.417	0.723	0.660	644
*RawMSA	0.677	0.428	0.237	0.281	0.455	0.586	0.763	646
*DISOPRED-3.1	0.664	0.416	0.289	0.262	0.429	0.620	0.711	646
Predisorder	0.664	0.422	0.237	0.260	0.433	0.567	0.763	642
IUPred2A-short	0.665	0.415	0.250	0.252	0.414	0.583	0.750	646
AUCpreD-np	0.662	0.425	0.269	0.251	0.423	0.592	0.731	646
fIDPnn	0.667	0.439	0.304	0.247	0.417	0.622	0.696	645
*SPOT-Disorder-Single	0.663	0.428	0.298	0.247	0.417	0.616	0.702	646
MobiDB-lite	0.654	0.407	0.227	0.244	0.420	0.538	0.773	645
IsUnstruct	0.656	0.417	0.268	0.243	0.414	0.581	0.732	646
ESpritz-X	0.666	0.425	0.316	0.240	0.391	0.642	0.684	645
IUPred2A-long	0.649	0.388	0.244	0.239	0.433	0.542	0.756	646
VSL2B	0.652	0.410	0.280	0.237	0.407	0.582	0.720	644
ESpritz-N	0.645	0.387	0.228	0.236	0.416	0.528	0.772	645
JRONN	0.638	0.384	0.208	0.234	0.431	0.495	0.792	645
fIDPln	0.645	0.406	0.290	0.219	0.421	0.566	0.710	645
DynaMine	0.638	0.386	0.252	0.219	0.397	0.537	0.748	645
DisoMine	0.637	0.408	0.364	0.207	0.401	0.620	0.636	646
PyHCA	0.636	0.383	0.300	0.201	0.382	0.570	0.700	646
**PDB Close**	**0.598**	**0.383**	**0.365**	**0.199**	**0.404**	**0.561**	**0.635**	**604**
**Gene3D**	**0.630**	**0.405**	**0.474**	**0.188**	**0.380**	**0.709**	**0.526**	**646**
DisEMBL-465	0.617	0.368	0.256	0.180	0.370	0.498	0.744	644
*S2D-2	0.616	0.362	0.383	0.172	0.338	0.617	0.617	644
**PDB Remote**	**0.601**	**0.338**	**0.412**	**0.171**	**0.343**	**0.614**	**0.588**	**523**
FoldUnfold	0.620	0.386	0.382	0.169	0.383	0.607	0.618	621
**PDB observed**	**0.609**	**0.428**	**0.507**	**0.164**	**0.408**	**0.697**	**0.493**	**646**
ESpritz-D	0.610	0.358	0.321	0.155	0.381	0.525	0.679	645
DisEMBL-HL	0.605	0.346	0.342	0.137	0.323	0.551	0.658	644
GlobPlot	0.592	0.338	0.381	0.136	0.325	0.572	0.619	645
**Conservation**	**0.571**	**0.330**	**0.585**	**0.111**	**0.306**	**0.719**	**0.415**	**646**
*DisPredict-2	0.560	0.319	0.484	0.058	0.293	0.579	0.516	646
DFLpred	0.532	0.348	0.996	0.000	0.257	0.995	0.004	646

Supplementary Table 4. Per-protein classification performance in the DisProt dataset. Performance of predictors and baselines for *DisProt* dataset. Metrics are averaged over the whole dataset, sorted by F1-Score and predictor thresholds are optimized on F1-Score. Baselines are shown in

bold and AlphaFold in italics and underlined. The evaluation metrics are as follows: balanced accuracy (BAC), F1-Score (F1-s), false positive rate (FPR); Matthews correlation coefficient (MCC), positive predictive value (PPV, i.e. precision), true positive rate (TPR, i.e. recall), true negative rate (TNR, i.e. specificity) and coverage (COV), i.e. number of predicted target proteins (out of 646).

DisProt-Binding dataset

	BAC	F1-s	FPR	MCC	PPV	TPR	TNR	COV
ANCHOR-2	0.665	0.231	0.217	0.189	0.146	0.548	0.783	646
AlphaFold-Bind	*0.617*	*0.222*	*0.123*	*0.164*	*0.161*	*0.357*	*0.877*	*489*
DisoRDPbind-protein	0.690	0.216	0.330	0.194	0.127	0.711	0.670	646
MoRFchibi-light	0.609	0.215	0.122	0.154	0.157	0.340	0.878	644
MoRFchibi-web	0.600	0.202	0.128	0.139	0.146	0.327	0.872	644
OPAL	0.610	0.200	0.165	0.139	0.135	0.385	0.835	644
**Gene3D**	**0.656**	**0.175**	**0.516**	**0.153**	**0.098**	**0.828**	**0.484**	**646**
DISOPRED-3.1-binding	0.569	0.169	0.125	0.099	0.124	0.263	0.875	646
**PDB observed**	**0.606**	**0.152**	**0.589**	**0.106**	**0.084**	**0.801**	**0.411**	**646**
fMoRFpred	0.526	0.125	0.587	0.026	0.069	0.639	0.413	646
DisoRDPbind-DNA	0.528	0.124	0.352	0.028	0.073	0.407	0.648	646
DisoRDPbind-RNA	0.499	0.119	0.998	-0.006	0.063	0.997	0.002	646
DisoRDPbind	0.500	0.119	1.000	0.000	0.063	1.000	0.000	646

Supplementary Table 5. Per-residue classification performance in the DisProt-Binding dataset. Performance of predictors and baselines for *DisProt-Binding* dataset. Metrics are averaged over the whole dataset, sorted by F1-Score and predictor thresholds are optimized on F1-Score. Baselines are shown in bold and AlphaFold in italics and underlined. The evaluation metrics are as follows: balanced accuracy (BAC), F1-Score (F1-s), false positive rate (FPR); Matthews correlation coefficient (MCC), positive predictive value (PPV, i.e. precision), true positive rate (TPR, i.e. recall), true negative rate (TNR, i.e. specificity) and coverage (COV), i.e. number of predicted target proteins (out of 646).

	BAC	F1-s	FPR	MCC	PPV	TPR	TNR	COV
**Gene3D**	**0.529**	**0.143**	**0.523**	**0.053**	**0.125**	**0.261**	**0.477**	**646**
DisoRDPbind-protein	0.663	0.131	0.334	0.059	0.142	0.215	0.666	646
DisoRDPbind	0.194	0.131	1.000	0.000	0.100	0.358	0.000	646
DisoRDPbind-RNA	0.195	0.131	0.999	-0.001	0.100	0.357	0.001	646
**PDB observed**	**0.490**	**0.128**	**0.569**	**-0.011**	**0.115**	**0.224**	**0.431**	**646**
OPAL	0.604	0.121	0.360	0.029	0.122	0.166	0.640	644
fMoRFpred	0.430	0.116	0.627	0.004	0.100	0.236	0.373	646
AlphaFold-Bind	*0.728*	*0.114*	*0.201*	*0.053*	*0.139*	*0.141*	*0.799*	*489*
ANCHOR-2	0.734	0.111	0.209	0.054	0.142	0.160	0.791	646
MoRFchibi-web	0.668	0.109	0.262	0.038	0.131	0.137	0.738	644
MoRFchibi-light	0.673	0.106	0.252	0.036	0.131	0.131	0.748	644
DISOPRED-3.1-binding	0.725	0.095	0.172	0.036	0.130	0.105	0.828	646
DisoRDPbind-DNA	0.588	0.092	0.369	0.002	0.100	0.143	0.631	646

Supplementary Table 6. Per-protein classification performance in the DisProt-Binding dataset. Performance of predictors and baselines for *DisProt-Binding* dataset. Metrics are averaged over the whole dataset, sorted by F1-Score and predictor thresholds are optimized on F1-Score. Baselines are shown in bold and AlphaFold in italics and underlined. The evaluation metrics are as follows: balanced accuracy (BAC), F1-Score (F1-s), false positive rate (FPR); Matthews correlation coefficient (MCC), positive predictive value (PPV, i.e. precision), true positive rate (TPR, i.e. recall), true negative rate (TNR, i.e. specificity) and coverage (COV), i.e. number of predicted target proteins (out of 646).

Fully Disordered Proteins

	TN	FP	FN	TP	MCC	F1-s	TNR	TPR	PPV	BAC
fIDPnn	458	8	7	16	0.665	0.681	0.983	0.696	0.667	0.839
SPOT-Disorder-Single	463	3	12	11	0.599	0.595	0.994	0.478	0.786	0.736
RawMSA	453	13	8	15	0.569	0.588	0.972	0.652	0.536	0.812
Predisorder	460	6	11	12	0.572	0.585	0.987	0.522	0.667	0.754
ESpritz-N	464	2	14	9	0.553	0.529	0.996	0.391	0.818	0.694
SPOT-Disorder2	448	18	9	14	0.488	0.509	0.961	0.609	0.438	0.785
IUPred2A-long	462	4	14	9	0.504	0.500	0.991	0.391	0.692	0.691
AUCpreD	459	7	13	10	0.485	0.500	0.985	0.435	0.588	0.710
DisoMine	437	29	6	17	0.491	0.493	0.938	0.739	0.370	0.838
IsUnstruct	458	8	13	10	0.470	0.488	0.983	0.435	0.556	0.709
VSL2B	448	18	11	12	0.426	0.453	0.961	0.522	0.400	0.742
IUPred2A-short	465	1	16	7	0.504	0.452	0.998	0.304	0.875	0.651
MobiDB-lite	465	1	16	7	0.504	0.452	0.998	0.304	0.875	0.651
fIDPln	444	22	10	13	0.425	0.448	0.953	0.565	0.371	0.759
AlphaFold-RSA	*427*	*39*	*6*	*17*	*0.436*	*0.430*	*0.916*	*0.739*	*0.304*	*0.828*
JRONN	463	3	16	7	0.446	0.424	0.994	0.304	0.700	0.649
DisPredict-2	462	4	16	7	0.422	0.412	0.991	0.304	0.636	0.648
ESpritz-X	464	2	17	6	0.428	0.387	0.996	0.261	0.750	0.628
ESpritz-D	435	31	11	12	0.340	0.364	0.933	0.522	0.279	0.728
S2D-2	443	23	13	10	0.325	0.357	0.951	0.435	0.303	0.693
**Gene3D**	**401**	**65**	**4**	**19**	**0.385**	**0.355**	**0.861**	**0.826**	**0.226**	**0.843**
PyHCA	464	2	18	5	0.380	0.333	0.996	0.217	0.714	0.607
**PDB observed**	**373**	**93**	**7**	**16**	**0.252**	**0.242**	**0.800**	**0.696**	**0.147**	**0.748**
FoldUnfold	358	108	6	17	0.246	0.230	0.768	0.739	0.136	0.754
**PDB Remote**	**455**	**11**	**19**	**4**	**0.185**	**0.211**	**0.976**	**0.174**	**0.267**	**0.575**
DISOPRED-3.1	462	4	20	3	0.217	0.200	0.991	0.130	0.429	0.561
DisEMBL-HL	466	0	21	2	0.288	0.160	1.000	0.087	1.000	0.543
DisEMBL-465	466	0	22	1	0.204	0.083	1.000	0.043	1.000	0.522
**Conservation**	**328**	**138**	**20**	**3**	**-0.077**	**0.037**	**0.704**	**0.130**	**0.021**	**0.417**
DFLpred	466	0	23	0	0.000	0.000	1.000	0.000	0.000	0.500
DynaMine	466	0	23	0	0.000	0.000	1.000	0.000	0.000	0.500
AlphaFold-pLDDT	*466*	*0*	*23*	*0*	*0.000*	*0.000*	*1.000*	*0.000*	*0.000*	*0.500*
GlobPlot	466	0	23	0	0.000	0.000	1.000	0.000	0.000	0.500
**PDB Close**	**456**	**10**	**23**	**0**	**-0.032**	**0.000**	**0.979**	**0.000**	**0.000**	**0.489**

Supplementary Table 7. Confusion matrix and metrics for the prediction of fully disordered proteins in the DisProt dataset.

Proteins with disorder prediction or disorder annotation covering at least 95% of the sequence are considered fully disordered. The subset of proteins predicted by AlphaFold are considered (n= 489). Predictors are sorted by their F1-score. Baseline names are in bold. The evaluation metrics are as follows: true negatives (TN(, true positives (TP), false negatives (FN), false positives (FP), F1-score (F1-s), true negative rate (TNR, i.e. specificity) true positive rate (TPR, i.e. recall), positive predictive value (PPV, i.e. precision), balanced accuracy for prediction of fully disordered proteins (BAC).

Pred	DisProt	UniProt	Protein name	Organism	Length	Content
TP	DP01135	P40019	Histone H2A.Z-specific chaperone CHZ1	*S. cerevisiae*	153	100%
TP	DP01159	A6NF83	Nuclear protein 2	*Homo sapiens*	97	100%
TP	DP01299	Q96273	Late embryogenesis abundant protein 18	*A. thaliana*	97	100%
TP	DP01300	Q9SLJ2	Dehydrin HIRD11	*A. thaliana*	98	100%
TP	DP01425	Q15004	PCNA-associated factor	*Homo sapiens*	111	100%
TP	DP01435	O60829	P antigen family member 4	*Homo sapiens*	102	100%
TP	DP01471	P36118	Pre-mRNA-splicing factor NTR2	*S. cerevisiae*	322	100%
TP	DP01521	Q5RJL0	Ermin	*R. norvegicus*	282	100%
TP	DP01559	Q04964	Ribonucleotide reductase inhibitor protein SML1	*S. cerevisiae*	104	100%
TP	DP01677	P06454	Prothymosin alpha	*Homo sapiens*	111	100%
TP	DP01776	Q8GYN5	RPM1-interacting protein 4	*A. thaliana*	211	100%
TP	DP01858	Q96270	Late embryogenesis abundant protein 7	*A. thaliana*	169	100%
TP	DP01876	P52926	High mobility group protein HMGI-C	*Homo sapiens*	109	100%
TP	DP02005	P47710	Alpha-S1-casein	*Homo sapiens*	185	100%
TP	DP02066	Q9FG31	Late embryogenesis abundant protein 46	*A. thaliana*	158	100%
TP	DP02078	P02686-5	Isoform 5 of Myelin basic protein	*Homo sapiens*	171	100%
TP	DP02205	Q2TUW1	Abscisic stress ripening-like protein	*Glycine max*	238	100%
FP	DP01448	P10923	Osteopontin	*Mus musculus*	294	94.56%
FP	DP01558	Q9XTY3	Seven B Two (Mammalian 7BT prohormone convertase chaperone) homolog	*C. elegans*	211	92.42%
FP	DP01557	P27682	Neuroendocrine protein 7B2	*R. norvegicus*	210	88.57%
FP	DP01173	Q15726	Metastasis-suppressor KiSS-1	*Homo sapiens*	138	86.23%
FP	DP01473	Q96GU1	P antigen family member 5	*Homo sapiens*	130	85.38%
FP	DP01502	P33328	Synaptobrevin homolog 2	*S. cerevisiae*	115	79.13%
FP	DP01126	O43561	Linker for activation of T-cells family member 1	*Homo sapiens*	262	78.63%
FP	DP01191	O14140	26S proteasome complex subunit rpn15	*S. pombe*	71	76.06%
FP	DP01453	Q8NHZ8	Anaphase-promoting complex subunit CDC26	*Homo sapiens*	85	70.59%
FP	DP01246	O13916	Anaphase-promoting complex subunit hcn1	*S. pombe*	80	70.00%
FP	DP01658	Q09882	Pre-mRNA-processing protein 45	*S. pombe*	557	61.22%
FP	DP01943	Q9NRY2	SOSS complex subunit C	*Homo sapiens*	104	59.62%
FP	DP01392	P53330	Regulator of Ty1 transposition protein 102	*S. cerevisiae*	157	54.78%
FP	DP01454	P60006	Anaphase-promoting complex subunit 15	*Homo sapiens*	121	53.72%
FP	DP01498	O70480	Vesicle-associated membrane protein 4	*Mus musculus*	141	50.35%
FP	DP01747	P48788	Troponin I, fast skeletal muscle	*Homo sapiens*	182	47.25%
FP	DP01281	Q9JM54	Phorbol-12-myristate-13-acetate-induced protein 1	*Mus musculus*	103	46.60%
FP	DP01455	Q96DE5	Anaphase-promoting complex subunit 16	*Homo sapiens*	110	46.36%

FP	DP02296	P60761	Neurogranin	*Mus musculus*	78	43.59%
FP	DP02269	Q9Y6K9	NF-kappa-B essential modulator	*Homo sapiens*	419	40.57%
FP	DP01172	O43516	WAS/WASL-interacting protein family member 1	*Homo sapiens*	503	39.17%
FP	DP01643	O75506	Heat shock factor-binding protein 1	*Homo sapiens*	76	35.53%
FP	DP01608	Q9Y6H6	Potassium voltage-gated channel subfamily E member 3	*Homo sapiens*	103	34.95%
FP	DP01883	P02655	Apolipoprotein C-II	*Homo sapiens*	101	25.74%
FP	DP02061	P70447	Neurogenin-2	*Mus musculus*	263	22.05%
FP	DP01203	O75807	Protein phosphatase 1 regulatory subunit 15A	*Homo sapiens*	674	17.66%
FP	DP01794	Q99ML1	Bcl-2-binding component 3	*Mus musculus*	193	17.62%
FP	DP01434	P31431	Syndecan-4	*Homo sapiens*	198	14.14%
FP	DP02170	O15234	Protein CASC3	*Homo sapiens*	703	12.94%
FP	DP01313	Q9N4U5	Smu-2 suppressor of mec-8 and unc-52 protein	*C. elegans*	547	11.15%
FP	DP02149	Q24570	Cell death protein Grim	*D. melanogaster*	138	10.87%
FP	DP01999	Q8K4J6	Myocardin-related transcription factor A	*Mus musculus*	964	9.96%
FP	DP01139	A0A0G2JXC5	Myocardin-related transcription factor A	*R. norvegicus*	1038	9.25%
FP	DP02122	Q02199	Nucleoporin NUP49/NSP49	*S. cerevisiae*	472	7.20%
FP	DP01603	Q96IZ0	PRKC apoptosis WT1 regulator protein	*Homo sapiens*	340	6.76%
FP	DP01994	P27321	Calpastatin	*R. norvegicus*	713	5.89%
FP	DP01366	Q24139	Protein commissureless 1	*D. melanogaster*	370	2.97%
FP	DP01278	Q68DK7	Male-specific lethal 1 homolog	*Homo sapiens*	614	1.79%

Supplementary Table 8. AlphaFold-RSA performance of predicted fully disordered proteins. The columns are (from left to right): AlphaFold-RSA prediction (Pred; TP: true positive, FP: false positive), DisProt and UniProt accession numbers, protein name and organism, length in amino acids, DisProt disorder content (Content).

Figures

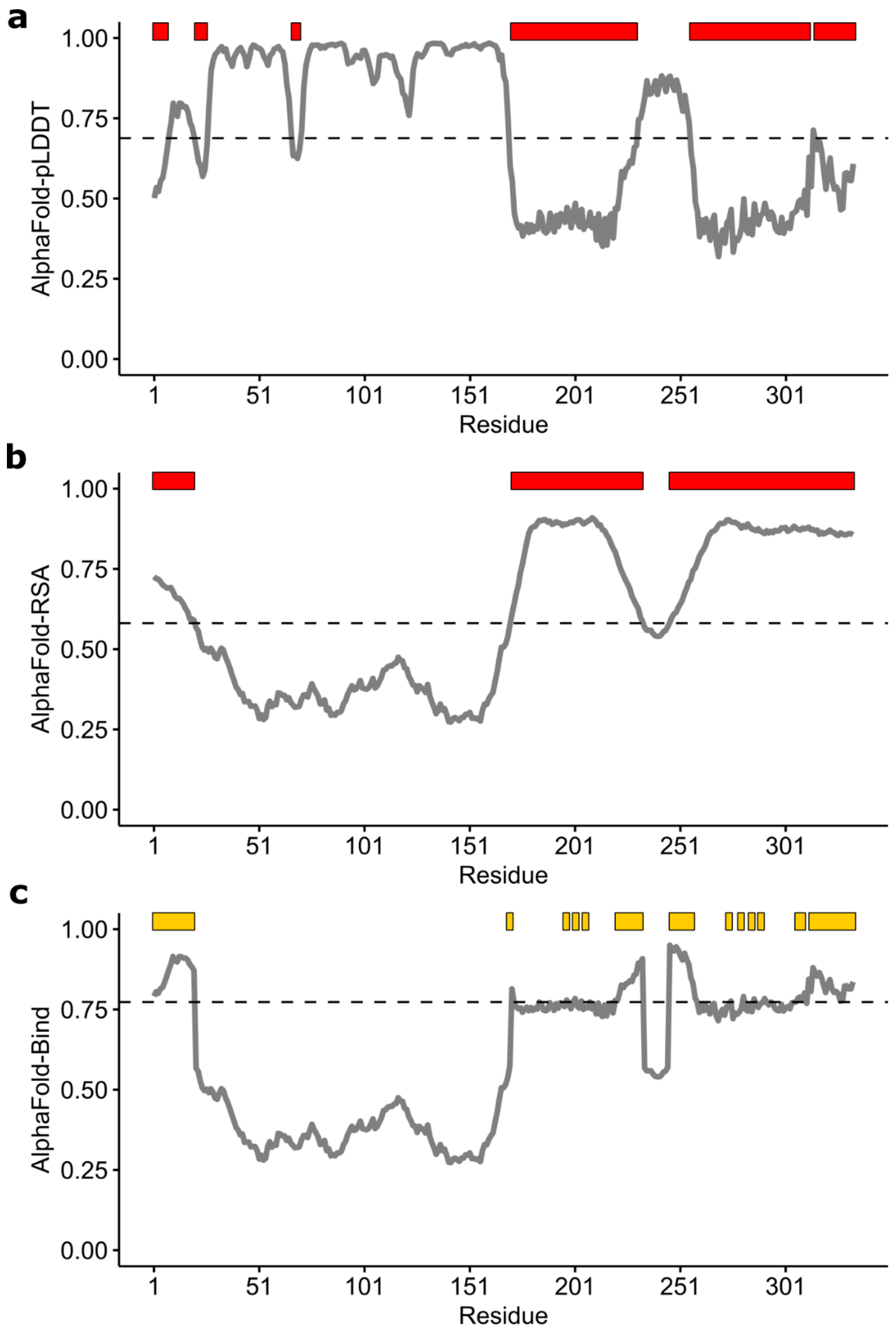

Supplementary Figure 1 - Intrinsic disorder and conditional folding per-residue scores derived from AlphaFold prediction.
AlphaFold-pLDDT (a), AlphaFold-RSA (b) and AlphaFold-Bind (c) scores and optimal thresholds for the human Ephrin-B2 protein (UniProt accession: P52799). Horizontal bars indicate sequence positions passing the threshold (dashed line). Optimal thresholds are 0.688 (AlphaFold-pLDDT), 0.581 (AlphaFold-RSA) and 0.773 (AlphaFold-Bind).
